# Effects of low-dose computed tomography on lung cancer screening: a systematic review, meta-analysis, and trial sequential analysis

**DOI:** 10.1186/s12890-019-0883-x

**Published:** 2019-07-11

**Authors:** Kai-Lin Huang, Shih-Yuan Wang, Wan-Chen Lu, Ya-Hui Chang, Jian Su, Yen-Ta Lu

**Affiliations:** 1 Department of Pharmacy, MacKay Memorial Hospital, No. 92, Sec. 2, Zhongshan N. Rd., Taipei City, 10449 Taiwan; 2 Mackay Junior College of Medicine, Nursing, and Management, No. 92, Shengjing Road, Beitou District, Taipei, 11272 Taiwan; 3 Department of Chest Medicine, MacKay Memorial Hospital, No. 92, Sec. 2, Zhongshan N. Rd., Taipei City, 10449 Taiwan; 4 Department of Medicine, Mackay Medical College, No. 46, Sec. 3, Zhongzheng Rd., Sanzhi Dist., New Taipei City, 252 Taiwan

**Keywords:** Low-dose computed tomography, LDCT, Lung cancer screening, Mortality, Meta-analysis

## Abstract

**Background:**

The Nelson mortality results were presented in September 2018. Four other randomized control trials (RCTs) were also reported the latest mortality outcomes in 2018 and 2019. We therefore conducted a meta-analysis to update the evidence and investigate the benefits and harms of low-dose computed tomography (LDCT) in lung cancer screening.

**Methods:**

Detailed electronic database searches were performed to identify reports of RCTs that comparing LDCT to any other type of lung cancer screening. Pooled risk ratios (RRs) were calculated using random effects models.

**Results:**

We identified nine RCTs (*n* = 97,244 participants). In pooled analyses LDCT reduced lung cancer mortality (RR 0.83, 95% CI 0.76–0.90, I^2^ = 1%) but had no effect on all-cause mortality (RR 0.95, 95% CI 0.90–1.00). Trial sequential analysis (TSA) confirmed the results of our meta-analysis. Subgroup defined by high quality trials benefitted from LDCT screening in reducing lung cancer mortality (RR 0.82, 95% CI 0.73–0.91, I^2^ = 7%), whereas no benefit observed in other low quality RCTs. LDCT was associated with detection of a significantly higher number of early stage lung cancers than the control. No significant difference (RR 0.64, 95% CI 0.30–1.33) was found in mortality after invasive procedures between two groups.

**Conclusions:**

In meta-analysis based on sufficient evidence demonstrated by TSA suggests that LDCT screening is superiority over usual care in lung cancer survival. The benefit of LDCT is expected to be heavily influenced by the risk of lung cancer in the different target group (smoking status, Asian) being screened.

**Electronic supplementary material:**

The online version of this article (10.1186/s12890-019-0883-x) contains supplementary material, which is available to authorized users.

## Background

Cancer is a leading cause of death worldwide, accounting for an estimated 9.6 million deaths in 2018 [1]. Lung cancer is the commonest form of cancer (2.09 million cases) as well as the main cause of cancer related mortality (1.76 million deaths) [1]. Due to the asymptomatic nature of lung cancer, they are often diagnosed at an advanced stage when the prognosis is poor or futile. In more recent years low-dose computed tomography (LDCT) has been demonstrated to be a sensitive tool for the detection of early stage lung cancer [2]. However, researches also indicated that LDCT is associated with high false-positive rates in the diagnosis of lung cancer, resulting in unnecessary invasive procedures and patient anxiety [3–5]. In 2011, a high quality trial, the National Lung Screening Trial (NLST) [6], compared LDCT to chest radiology (CXR) in a large sample of high risk adults showed a 20% relative reduction in lung cancer mortality for LDCT over 6.5 years. Since then, lung cancer screening using LDCT for high risk groups is recommended by lots of organizations. But the most recent meta-analysis did not demonstrate superiority of LDCT screening over usual care in lung cancer mortality [7, 8].

Systematic reviews of randomized controlled trials (RCTs) are well recognized as the most reliable and appropriate reference standard to address questions of various types of medical intervention. In September 2018, new data from the largest European trial (NELSON) showed an even bigger reduction in deaths from lung cancer than was seen in NLST [9]. There were also four other RCTs [10–13] reported the mortality results in 2018 and 2019. Moreover, trial sequential analyses (TSA) of LDCT for lung cancer screening have not been reported previously. We aim to assess the updated evidence regarding the ability of LDCT to reduce lung cancer mortality and to evaluate the possible harms associated with LDCT screening.

## Methods

This review and meta-analysis was reported according to the Preferred Reporting Items for Systematic Reviews and Meta-Analysis (PRISMA) guidelines [14]. The protocol was registered with the International Prospective Registry of Systematic Reviews (PROSPERO at www.crd.york.ac.uk under following ID: CRD42018111630).

### Search strategy

The databases searched for this study were composed of Medline (Ovid), EMBASE, CENTRAL (Cochrane Database of Systematic Reviews), CINAHL (Cumulative Index to Nursing and Allied Health Literature), Index to Taiwan Periodical Literature System, TRIP Database and Google Scholar (all from inception until June 17, 2019). Reference lists of the selected studies and systematic reviews were further reviewed for additional citations of published or unpublished reports. Automatic e-mail updates for saved searches were set up to identify new search results from the databases.

The search strategy consisted of subject headings, keywords and related terms for these topics. Language restrictions were not used. The MEDLINE (Ovid) search strategy can be found in Additional file 1: Table S1.

### Eligibility criteria

We included studies that met all of the following criteria: (1) we accepted only randomized controlled trials; (2) comparing LDCT to any other type of lung cancer screening; (3) adults, aged≧18 years, asymptomatic with risk factor for lung cancer (current or former smokers, family history of lung cancer, underlying lung disease, or environmental exposure to toxins); (4) benefits of interest included: lung cancer mortality, all-cause mortality, early detection (stage I) rates; (5) harms of interest included: death and major complications after invasive procedures (30–60 days post invasive procedures). Major complications were listed below: death, anaphylaxis, cardiac arrest, cerebral vascular accident/stroke, congestive heart failure, myocardial infarction, intervention-required thromboembolic complications, acute respiratory failure, respiratory arrest, bronchial stump leak requiring tube thoracostomy or other drainage for > 4 days, bronchopulmonary fistula, empyema, prolonged mechanical ventilation > 48 h postoperatively, tube placement-required hemothorax, brachial plexopathy, lung collapse, chylous fistula, injury to vital organ or vessel, wound dehiscence, and infarcted sigmoid colon. Invasive procedures included: surgery, biopsy, bronchoscopy or fine needle aspiration cytology.

Two independent authors (KLH and SYW) screened the trials based on the above criteria, and disagreements were resolved by consultation with a third author (WCL). Included studies were then assessed for methodological quality using the revised Cochrane risk-of-bias tool for randomized trials (RoB 2) [15]. The assessed factors included risk of bias arising from the randomization process, risk of bias due to deviations from the intended interventions, missing outcome data, risk of bias in measurement of the outcome and risk of bias in selection of the reported result.

### Data extraction

KLH and SYW extracted the data respectively, with disagreements resolved by consultation with other team members. Data related to the study characteristics and outcomes were collected from included trials. We extracted the following data: study name, country, number of participants, characteristics of population, screening type and interval, definition of positive results and outcome measures.

### Statistical analysis

We carried out analysis using Review Manager (RevMan) Version 5.3 (Copenhagen: The Nordic Cochrane Centre, The Cochrane Collaboration, 2014) software. Inverse variance meta-analysis for combining data was performed. If clinical or methodological heterogeneity between the study results was suspected, a random-effects meta-analysis was used. Results as summary risk ratio (RR) with 95% confidence intervals (CI) for dichotomous data were presented. Statistical heterogeneity was assessed using the Tau^2^, I^2^ and Chi^2^ statistics. We considered heterogeneity as substantial if the Tau^2^ was greater than zero, or there was a low *P* value (less than 0.10) in the Chi^2^ test for heterogeneity. For the I^2^ metric, moderate, substantial and considerable heterogeneity were considered to be 30–60%, 50–90%, and 75–100%, respectively. The following subgroup analyses were also conducted based on: (1) type of control groups (such as CXR screening or usual care or no screening); (2) quality of studies; (3) sample size; (4) sex. Sensitivity analyses were performed to examine the robustness of the effect size. The funnel plot approach was used to investigate publication bias if we included more than 10 studies in the analysis of the outcome in question. A trial sequential analysis was conducted using software (TSA Viewer, version 0.9.5.10 Beta). This is a type of cumulative meta-analysis that reduces both type I and type II errors from repetitive statistical testing. Trial sequential analysis provides the necessary sample size for our meta-analysis and boundaries that determine whether the evidence in our meta-analysis is reliable and conclusive [16, 17]. The required information size was calculated, and the trial sequential monitoring boundaries were computed using the O’Brien-Fleming approach. An optimal information size was considered as a 2-sided 5% risk of a type I error, 20% risk of a type II error (power of 80%), relative risk reduction of 20%, and the pooled control group event rate across the included studies.

## Results

### Study selection

**Fig. 1:**
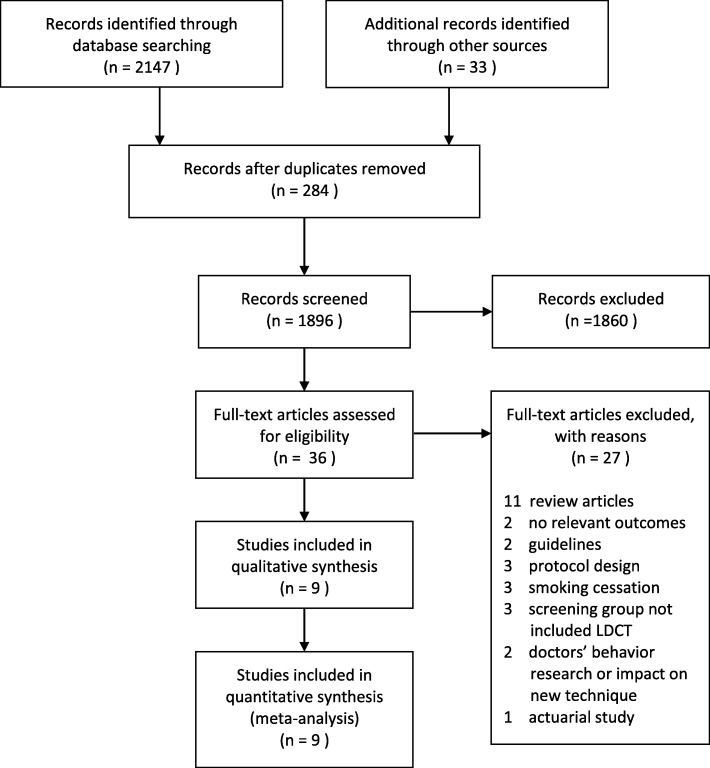
PRISMA (Preferred Reporting Items for Systematic Reviews and Meta-Analyses) diagram of study flow

Our literature search identified 2180 potentially relevant articles. Once duplicates had been removed, 1896 citations were screened, of which 36 full-text manuscripts were assessed for eligibility. Twenty-seven studies were excluded for the following reasons: eleven because they were review articles; two because there were no relevant outcomes (mortality data); two because they were guidelines; three because they were protocol designs; three because they were smoking cessation programs; three because the screening groups didn’t include LDCT; two because they interested in doctors’ behavior or impact on new technique; and one because it was an actuarial study. A list of full-text manuscripts that were excluded along with reasons for their exclusions is given in Additional file 2: Table S2. Finally, nine RCTs (with multiple publications) met our inclusion criteria. Figure 1 summarizes the literature search flow.

### Study characteristics

**Fig. 2:**
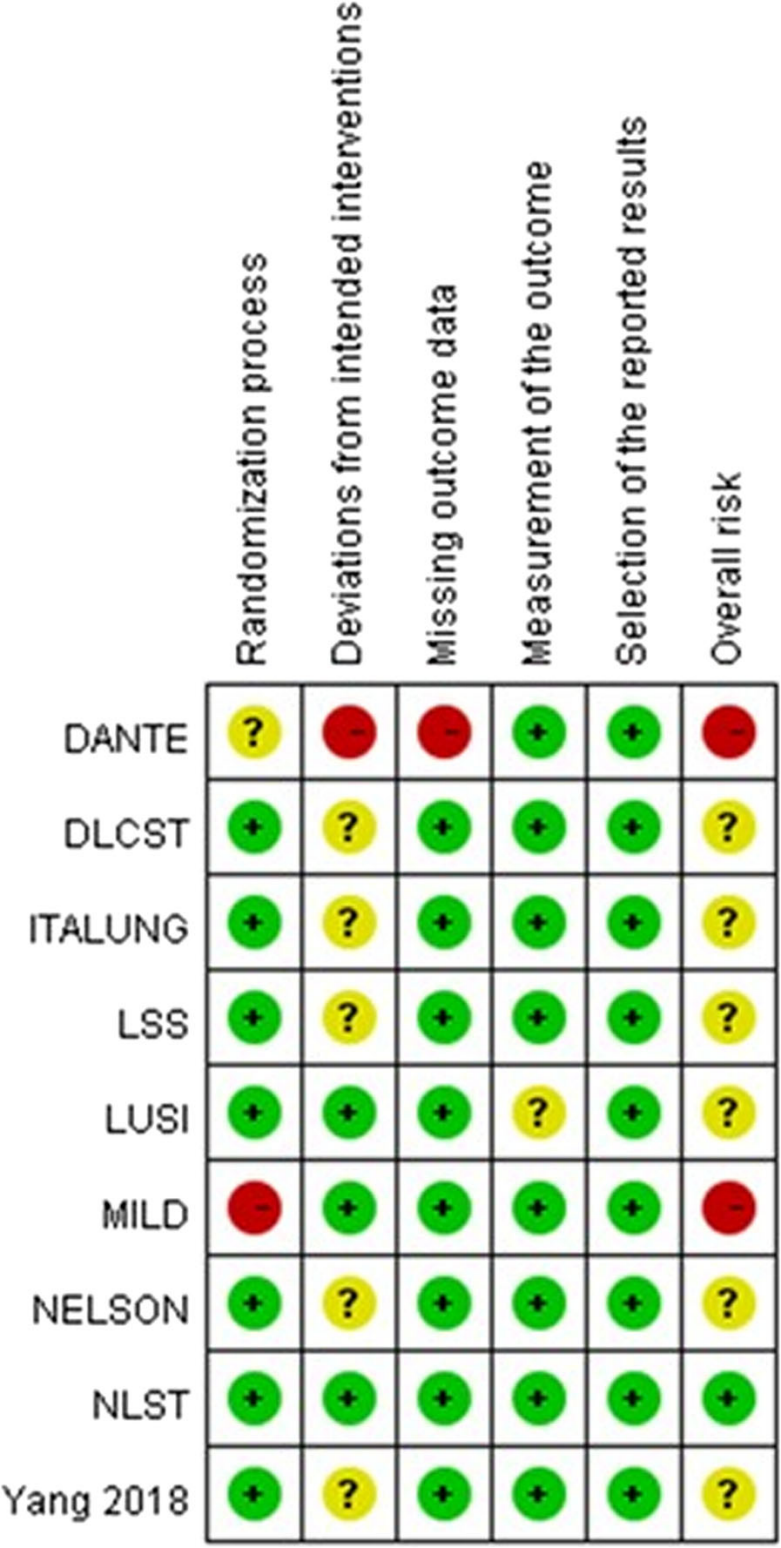
Risk of bias summary for included studies reporting mortality (red shading denotes high risk of bias, yellow shading denotes some concerns and green denotes low risk of bias)

Tables 1 and 2 presented the characteristics of selected studies. Seven trials [9, 10, 12, 13, 18–20] compared LDCT screening to no screening or usual care, and two trials [11, 21] compared LDCT to CXR. The trials were conducted in Italy, Denmark, Germany, USA, Netherlands, Belgium and China. Trials started between 2001 and 2013. One study [20] recruited only male. One study [10] had published preliminary mortality data and the duration of follow-up was less than 5 years. The sample size of included trials ranged from 2472 to 53,454. Most trials adopted 1 to 2-year interval screening. All included trials recruited high risk populations with age ranging from 45 to 75 years. The nature of high risk participants varied but was usually defined in terms of age and current and past smoking. Overall risk of bias for mortality outcomes was rated high in two RCTs [12, 20] and some concerns in six RCTs [9–11, 13, 18, 19]. Low overall risk rating was applied to one trial [21]. Domain ratings for each RCT are shown in Fig. 2.

**Table 1:** Summary screening criteria of included randomized controlled trials

Trials (country, start year)	No. randomized	Age (years)	Smoking history	Smoking cessation (years since quit)	Screening tests	Screening interval	Definition of positive results	Follow-up (years)
DANTE (Italy, 2001)	2472	60–74	≧20 pack-years	< 10	LDCT vs no screening	5 annual screens	> 5 mm	8.35 (median)
DLCST (Denmark, 2004)	4104	50–70	≧20 pack-years	< 10	LDCT vs no screening	5 annual screens	> 15 mm or rapid growing 5–15 mm nodules (> 25% increase in volume on 3-mo repeat CT	10
ITALUNG (Italy, 2004)	3206	55–69	≧20 pack-years	≦10	LDCT vs no screening	4 annual screens	≧5 mm solid nodule, a ground glass nodule ≧10 mm, or any part-solid nodule	8.5 (median)
LSS (USA, 2000)	3318	55–74	≧30 pack-years	< 10	LDCT vs CXR	1 screening	≧4 mm	5.2 (median)
LUSI (Germany,2007)	4052	50–69	≧15 cigarettes/day for ≧25 years or ≧10 cigarettes/day for ≧30 years	< 10	LDCT vs no screening	5 annual screens	≧5 mm	8.8
MILD (Italy, 2005)	4099	49–75	≧20 pack-years	< 10	LDCT vs no screening	5 annual screens or 3 biennial screens	Volume > 250 mm^3^ or rapid growing 60–250 mm^3^ (> 25% increase in volume on 3-mo repeat CT)	10
NELSON (Netherlands, Belgium, 2003)	15,822	50–75	≧15 cigarettes/day for ≧25 years or ≧10 cigarettes/day for ≧30 years	< 10	LDCT vs no screening	4 screening rounds; interval after baseline: 1y, 2y, and 2.5y	Volume > 500 mm^3^ or volume 50-500 mm^3^ with VDT < 400 d on 3-mo repeat CT	10
NLST (USA, 2002)	53,454	55–74	≧30 pack-years	≦15	LDCT vs CXR	3 annual screens	≧4 mm	7.4
Yang 2018 (China, 2013)	6717	45–70	≧20 pack-years (smoking is an optional risk factor)	≦15	LDCT vs no screening	3 biennial screens	≧4 mm	2

*Abbreviations*: *CXR* Chest radiography, *DANTE* Detection and screening of early lung cancer by novel imaging technology and molecular essays trial, *DLCST* Danish lung cancer screening trial, *ITALUNG* Italian lung cancer screening trial, *LDCT* Low-dose computed tomography, *LSS* Lung screening study, *LUSI* German lung cancer screening intervention trial, *MILD* Multi-centric Italian lung detection trial, *NELSON* Nederlands–Leuvens Longkanker screenings Onderzoek study, *NLST* National lung screening trial, *VDT* Volume doubling time

**Table 2:** Results of included randomized controlled trials

Trials (country, start year)	No. randomized	Age (y) [mean ± SD or median (IQR)]	Male (%)	Active smokers (%)	Pack-years [mean ± SD or median (IQR)]	Lung cancer mortality [RR (95% CI)]	All-cause mortality [RR (95% CI)]	Early detection (stage I) rates [RR (95% CI)]	Major complications^a^ after invasive procedures^b^ [RR (95% CI) or death number / total number of procedures]	Death after invasive procedures^b^ [RR (95% CI) or event number / total number of procedures]
DANTE (Italy, 2001)	2472	64.0 (5)	100	56.9	45.0 (28.5)	1.01 (0.70–1.44)	0.96 (0.79–1.16)	2.03* (1.26–3.29)	NR	0.75 (0.07–8.02)
DLCST (Denmark, 2004)	4104	57.9 ± 4.8	55.2	76.1	36.4 ± 13.4	1.03 (0.66–1.60)	1.01 (0.82–1.25)	3.31* (1.70–6.46)	LDCT: 4/49	LDCT: 0/49
ITALUNG (Italy, 2004)	3206	60.9	64.7	64.8	40 (NR)	0.71 (0.48–1.04)	0.84 (0.69–1.03)	3.18* (1.54–6.58)	NR	LDCT: 6/76
LSS (USA, 2000)	3318	Range: 55–74	58.6	57.5	54 (NR)	1.23 (0.74–2.05)	1.20 (0.94–1.52)	1.19* (0.63–2.22)	NR	NR
LUSI (Germany,2007)	4052	58.0 (5)	64.7	61.9	36 (18)	All: 0.72 (0.45–1.16) Female: 0.31* (0.10–0.94) Male: 0.92 (0.54–1.58)	0.98 (0.79–1.22) Female: 0.82 (0.47–1.42) Male: 1.02 (0.80–1.29)	NR	NR	NR
MILD (Italy, 2005)	4099	Annual: 57 (NR) Biennial: 58 (NR)	66.3	77.5	Annual: 39 (NR) Biennial: 39 (NR)	0.73 (0.47–1.12)	0.94 (0.73–1.20)	2.31* (1.37–3.88)	NR	NR
NELSON (Netherlands, Belgium, 2003)	15,822	58.0 (8)	83.6	55.4	38.0 (19.8)	Female: 0.61 (0.35–1.06) Male: 0.74* (0.60–0.91)	NR	NR	NR	NR
NLST (USA, 2002)	53,454	61 ± 5	59	48.2	48 (27)	0.85* (0.75–0.96)	0.94 (0.88–1.00)	1.32* (1.15–1.52)	1.30 (0.84–2.04)	0.60 (0.27–1.31)
Yang 2018 (China, 2013)	6717	59.8 ± 5.8	46.8	21.5	12.8 ± 17.2 (M) 9.1 ± 10.7 (F)	0.18 (0.01–3.72)	NR	4.71* (1.36–16.29)	NR	LDCT: 0/60

*Abbreviations*: *CI* Confidence interval, *IQR* Interquartile range, *NR* Not reported, *RR* Risk ratio, *SD* Standard deviation; see Table 1 legends for expansion of other abbreviations

*: Statistically significant differences

^a^Major complications included: death, anaphylaxis, cardiac arrest, cerebral vascular accident/stroke, congestive heart failure, myocardial infarction, intervention-required thromboembolic complications, acute respiratory failure, respiratory arrest, bronchial stump leak requiring tube thoracostomy or other drainage for > 4 days, bronchopulmonary fistula, empyema, prolonged mechanical ventilation > 48 h postoperatively, tube placement-required hemothorax, brachial plexopathy, lung collapse, chylous fistula, injury to vital organ or vessel, wound dehiscence, and infarcted sigmoid colon

^b^Invasive procedures included: surgery, biopsy, bronchoscopy or fine needle aspiration cytology

### Benefits and adverse outcomes

**Fig. 3:**
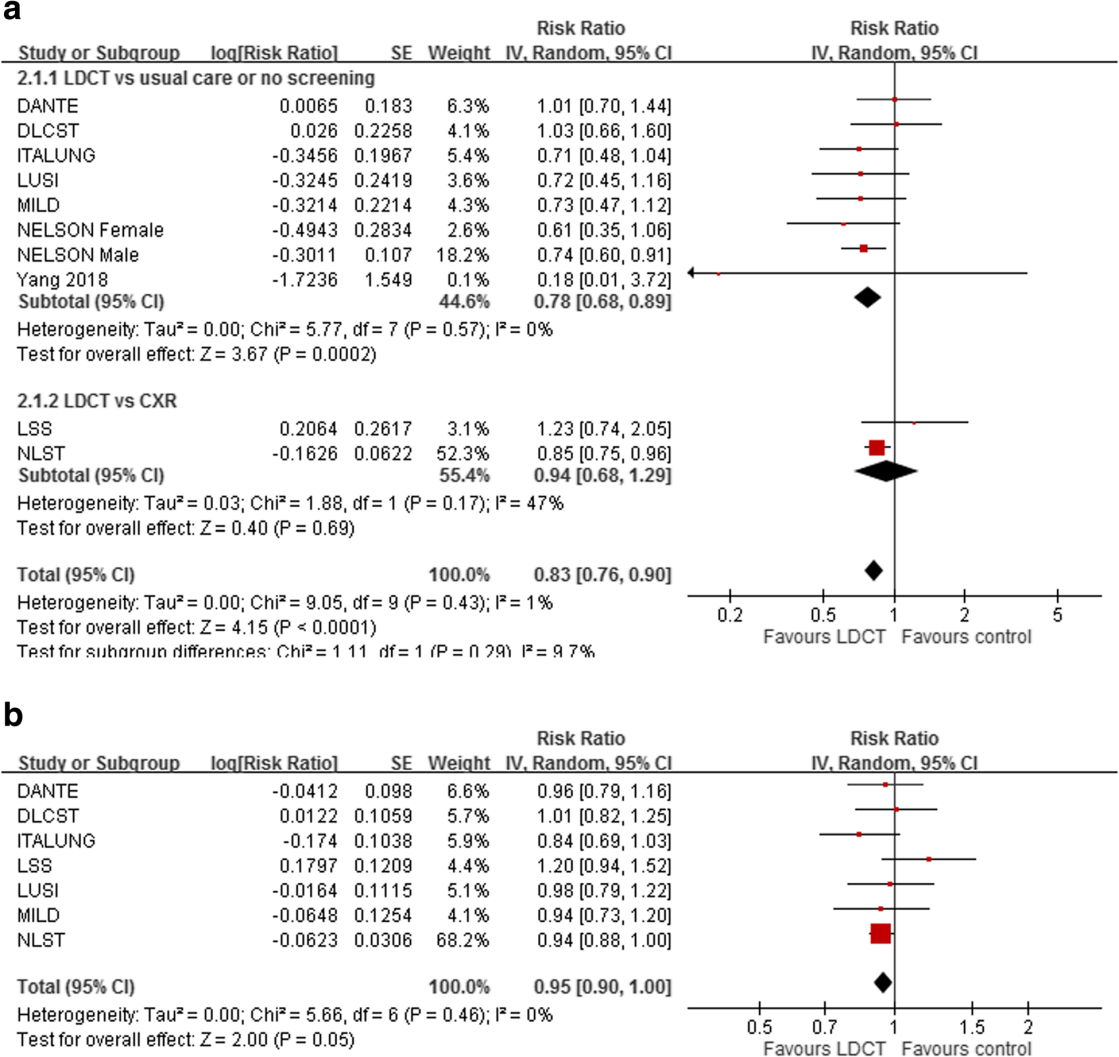
Forest plots of comparisons between low-dose computed tomography (LDCT) versus no screening or chest radiology (CXR) for **a** lung cancer mortality **b** all-cause mortality

There were nine [9–13, 18–21] contributing included studies to lung cancer mortality outcomes. When compared with controls (no screening or CXR), LDCT screening was associated with a statistically significant reduction in lung cancer mortality (RR 0.83, 95% CI 0.76–0.90) with no heterogeneity observed (*p* = 0.43, I^2^ = 1%; see Fig. 3a). Trial sequential analysis confirmed that the conclusion for lung cancer mortality was sufficient and no more trials were needed (Additional file 3: Figure S1). Seven included trials [11–13, 18–21] contributed information on all-cause mortality. On the contrary, LDCT screening demonstrated no statistically significant difference in all-cause mortality (RR 0.95, 95% CI 0.90–1.00) (Fig. 3b). There was no heterogeneity with this outcome (I^2^ = 0%). Pooled analysis of seven RCTs showed significantly greater proportions (RR 2.08, 95% CI 1.43–3.03) of early stage cancers in LDCT groups compared to controls.

As to the harm of screening, two studies reported number of death after invasive procedures for diagnosis purpose [6, 20]. Nineteen deaths were reported after 2129 invasive procedures in persons screened by LDCT and 11 deaths were reported after 792 invasive procedures in the control group. No significant difference (RR 0.64, 95% CI 0.30–1.33¸ I^2^ = 0) was shown. Only one study (NLST) reported major complication rates following invasive procedures for LDCT and CXR group. The risk was higher among persons who underwent LDCT compared with CXR screening (4.1 vs 3.2 per 10,000 screened) [6].

### Subgroup and sensitivity analyses

The role of subgroup and sensitivity analyses is to explore the potential sources of observed heterogeneity (Table 3). Among nine RCTs, the DANTE and MILD trials were judged to be of low quality (high risk of bias), whereas the remaining trials were judged to be of moderate to high quality (some concerns and low risk of bias; see Fig. 2). In the subgroup analysis according to study quality, compared with controls, LDCT screening demonstrated a statistically significant reduction in lung cancer mortality among high quality studies (RR 0.82, 95% CI 0.73–0.91). However, the same situation has not been observed in low quality studies (RR 0.87, 95% CI 0.64–1.20, I^2^ = 23%). As mentioned above, these suggest that trial quality might be a potential source of heterogeneity. We further explore the heterogeneity on the basis of sample size. We conducted a subgroup analysis based on the different sample size. A sample size that is too small reduces the power of the trial and increases the margin of error, which can render the trial meaningless. Pooled analysis of findings from seven [10–13, 18–20] fairly small trials (total *n* = 27,968) comparing LDCT with controls showed no significant difference in lung cancer mortality. While findings from two [9, 21] large trials (NELSON, NLST; total *n* = 69,276), the results of the pooled data displayed a RR of 0.80 (95% CI 0.71–0.91). In addition, regardless male or female, LDCT showed a reduction of lung cancer mortality. Sensitivity analyses were robust. The positive association was consistent with any of these analyses. Reliability and stability of our conclusions were further confirmed.

**Table 3:** Exploration of heterogeneity on the LDCT versus control for lung cancer mortality

Category	No. of estimates	Pooled RR (95% CI)	Heterogeneity I^2^ (%)
Total	9	0.83* (0.76–0.90)	1
Subgroup analyses			
Type of control groups			
LDCT versus no screening	7	0.78* (0.68–0.89)	0
LDCT versus CXR	2	0.94 (0.68–1.29)	47
Quality of studies			
Moderate to high quality	7	0.82* (0.73–0.91)	7
Low quality	2	0.87 (0.64–1.20)	23
Sample size			
Smaller size (DANTE, DLCST, ITALUNG, LUSI, MILD, Yang 2018, LSS)	7	0.87 (0.73–1.04)	5
Larger size (NELSON, NLST)	2	0.80* (0.71–0.91)	13
Sex			
Male	2	0.76* (0.63–0.93)	0
Female	2	0.52* (0.29–0.92)	13
Sensitivity analysis			
Exclusion of the studies from Asia and ≦ 5 years of follow up			
Exclude Yang 2018	8	0.83* (0.76–0.91)	1
Exclusion of studies in random manner			
Exclude DANTE	8	0.82* (0.74–0.89)	0
Exclude DLCST	8	0.82* (0.75–0.90)	1
Exclude ITALUNG	8	0.83* (0.75–0.92)	5
Exclude LUSI	8	0.83* (0.75–0.92)	8
Exclude MILD	8	0.83 * (0.75–0.92)	8
Exclude NELSON	8	0.86* (0.77–0.95)	0
Exclude LSS	8	0.82* (0.75–0.89)	0
Exclude NLST	8	0.81* (0.70–0.93)	7

See Table 1 legends for abbreviations

*Statistically significant differences

## Discussion

This is the first meta-analysis of LDCT for lung cancer screening based on sufficient evidence demonstrated by TSA with the latest NELSON, MILD and LUSI mortality results [9, 12, 13] included. NELSON trial is the only European fully powered RCT which presented its 10 year mortality findings in September 2018 at the International Association for the Study of Lung Cancer (IASLC) 19th World Conference on Lung Cancer (WCLC). In total, nine RCTs are included. Most RCTs (DANTE, DLCST, ITALUNG, LUSI, MILD, NELSON) are conducted in European countries, some trials are conducted in the USA (LSS, NLST) and China (Yang 2018). The majority of included studies are judged to be of moderate to high quality (some concerns and low risk of bias for mortality outcomes), but two studies (DANTE, MILD) are judged to be of low quality (high risk of bias for mortality outcomes). Pooled results comparing LDCT to no screening or CXR establish a survival benefit and show an increase in detection of stage I cancers. As for harms of lung cancer screening, LDCT leads to an increase in the frequency of invasive procedures, but does not lead to more death soon after an invasive procedure compared with the control arms. Our results are similar to previous meta-analyses [7, 8, 22, 23] but we identify more studies [9–11], more participants and more events which enhanced the precision of the results. We also conducted trial sequential analyses which provide estimates about the reliability of current evidence and prevent premature conclusions from meta-analyses.

A range of potential sources for heterogeneity is investigated. There is significant difference in the lung cancer mortality between subgroups of higher versus lower quality trials (*higher quality trials* RR 0.82 [95% CI 0.73–0.91] vs *lower quality trials* RR 0.87 [95% CI 0.64–1.20]). When removing the poor quality trial (DANTE and MILD), analysis reveal a significant decrease in lung cancer mortality in favor of LDCT compared with controls. High risk of bias ratings are applied to DANTE and MILD trials in our study. MILD trial has different LDCT screening strategies (annual and biennial). But recruitment (4099 subjects) is low, well below the announced sample size calculation (10,000 subjects, 30% mortality reduction at 10 years) and pronounced imbalances in baseline characteristics in three important characteristics (sex, current smoking status and predicted FEV1) are found. As for DANTE, the uneven numbers between LDCT (*n* = 1264) and control (*n* = 1186) groups seem not compatible with 1:1 scheme randomization in blocks of four mentioned in the methods. Apart from that, different randomized numbers in the publications from 2009 (*n* = 1276, 1196) [24] and 2015 (*n* = 1264, 1186) [20] raise question about the quality. The lack of precision on the methods of DANTE and MILD trials makes it difficult to interpret the results. A considerable reduction in heterogeneity is observed after we exclude the poor quality trial, suggesting that variation in trial quality may be a potential source of heterogeneity.

Sample size (statistical power of the trial) may also be a potential source of heterogeneity. Significant difference in the lung cancer mortality between subgroups of larger versus smaller trials (*larger trials* RR 0.80 [95% CI 0.71–0.91] vs *smaller trials* RR 0.87 [95% CI 0.73–1.04]) is observed. The two larger (15,822–53,454 subjects) trials (NLST, NELSON) have dominated the positive screening effects of the meta-analysis. NLST and NELSON are the only two trials that are powered enough for the outcome of lung cancer mortality. They are also the only two trials reported a significant decrease in lung cancer mortality. Seven smaller (2472–6717 subjects) trials (DANTE, DLCST, ITALUNG, LUSI, MILD, Yang 2018 and LSS) are not sufficiently powered to detect statistically significant differences in mortality and found no significant difference between the screening modalities due to the larger 95% CI.

In addition, there are major geographic differences, particularly in Asia, where 60 to 80% of women with lung cancer are never-smokers [25]. In Yang 2018 study [10], they enrolled fewer active smokers (21.5%) and males (46.8%) than other USA and European trials. Although smoking is the primary etiologic factor responsible for lung cancer, racial/ethnic and sex differences may exist. According data from WHO [26], age-standardized rate of current tobacco smoking among population aged≧15 years were estimated 2.2% for female in South-East Asia. Whereas for female in Americas and Europe, the rate were 12.4 and 20.7%. Previous studies [27] also indicated that lung cancer significantly associated with Asian non-smoking women. This group of lung cancers may be caused by other carcinogens rather than those contained in cigarettes. Only 7.1% participants in the LDCT group meet the NLST criteria in Yang 2018 study. If only western-eligible subjects receive LDCT screening, non-smoking-related lung cancer, which is highly prevalent in Asian women, will not be successfully identified. There is insufficient evidence to ascertain if USA and European criteria are appropriate for lung cancer screening programs outside of USA and Europe. There are some concerns about the quality of the trial. The uneven numbers between LDCT (*n* = 3550) and control (*n* = 3167) groups seem not compatible with 1:1 scheme randomization in blocks of 12 mentioned in the methods. Then the duration of follow-up is not sufficient (2 years) for now and probably do not have enough power to test the hypothesis out.

Several biases arise in the evaluation of screening studies, including lead-time, length-time and overdiagnosis, which should be taken into account when interpreting these data. Firstly, when lead time is short, as is true with lung cancer, it is difficult to demonstrate that treatment of medical condition found on screening is more effective than treatment after symptoms appear. Secondly, Screening is more likely to detect slow-growing tumors, which have a better prognosis, including longer survival. However, most type of cancers demonstrate a wide range of growth rates. Thirdly, although LDCT shows significantly greater proportions of early stage lung cancer compares to controls, further evaluation will be required to determine which patients with positive screening results have cancer. Higher early stage detection rates of LDCT not only results in excess follow-up testing but also psychological distress. Various trials [3, 28–30] suggest that LDCT screening has the potential to cause short-term (< 6 months after screen) psychosocial impact on high-risk participants but that effects do not appear to persist long term (> 6 months after screen). Overdiagnosis also results in unnecessary diagnostic procedures and lead to unnecessary treatment. The magnitude of overdiagnosis of LDCT was 18.5% (95% CI 5.4–30.6%) in NLST [28], 67.2% (95% CI 37.1–95.4%) in DLCST [29] and zero in ITALUNG [18]. It is important to note that the definition of overdiagnosis varied across studies. Finally, we generalize individual trials into groups although population, smoking history, number of screening rounds, duration of follow-up, definition of positive lung nodules, and radiologists’ skill may differ between trials. Caution is needed in interpreting the findings from our results.

## Conclusion

The present meta-analysis based on sufficient evidence demonstrated by TSA indicates that there is significant reduction in lung cancer mortality between LDCT and other control groups. Moreover, the results of the subgroup analyses indicate that, LDCT screening has shown statistically significant mortality benefits in high-quality trials, whereas low-quality trials found no significant difference. It is mandatory to identify lung cancer risk factors among the Asian population and to establish appropriate eligible criteria in the screening program for different races. The benefit of LDCT is expected to be heavily influenced by the risk of lung cancer in the different target group (smoking status, female and Asian) being screened. Due to tenuous balance of benefits and harms, medical decision making is recommended for individuals who are considering LDCT screening. More studies are warranted to optimize the approach to LDCT screening.

## Additional files

Additional file 1: **Table S1.** MEDLINE (Ovid) search strategy. (DOCX 14 kb)

Additional file 2: **Table S2.** Excluded studies with reasons. (DOCX 18 kb)

Additional file 3: **Figure S1.** Trial sequential analysis for lung cancer mortality. (DOCX 28 kb)

## Data Availability

All data generated or analyzed during this study are included in this published article and its supplementary information files.

## Abbreviations

CI: Confidence interval
CXR: Chest radiography
DANTE: Detection and Screening of Early Lung Cancer by Novel Imaging Technology and Molecular Essays Trial
DLCST: Danish Lung Cancer Screening Trial
IQR: Interquartile range
ITALUNG: Italian Lung Cancer Screening Trial
LDCT: Low-dose computed tomography
LSS: Lung Screening Study
LUSI: German Lung Cancer Screening Intervention Trial
MILD: Multi-centric Italian Lung Detection Trial
NELSON: Nederlands–Leuvens Longkanker Screenings Onderzoek Study
NLST: National Lung Screening Trial
RCTs: Randomized control trials
RR: Risk ratio
SD: Standard deviation
TSA: Trial sequential analysis
