# Supplementary material for: Effects of low-dose computed tomography on lung cancer screening: a systematic review, meta-analysis, and trial sequential analysis
Source: BMC Pulm Med. 2019 Jul 11;19:126. doi: 10.1186/s12890-019-0883-x (PMC6625016; doi:10.1186/s12890-019-0883-x)
Supplement: Supplementary file 1 — Table S1. MEDLINE (Ovid) search strategy. (DOCX 14 kb) [file 12890_2019_883_MOESM1_ESM.docx]

**Table S1.** MEDLINE (Ovid) search strategy

| # | Search terms |
| --- | --- |
| 1 | exp. lung neoplasms/lung neoplasm*.ti,ab. |
| 2 | ((lung* or bronch* or pulmon*) adj3 (cancer* or neopla* or tumor* or tumour* or carcinoma* or adenocarcinoma* or small cell or squamous)).ti,ab,ot,kw. |
| 3 | (NSLC or NSCLC or SLC or SCLC).ti,ab,ot,kw. |
| 4 | exp. tomography, x-ray computed/ |
| 5 | ((CT or CAT) adj3 (scan* or screen*)).ti,ab,ot,kw. |
| 6 | ((computer* adj3 tomogra*) and (scan* or screen*)).ti,ab,ot,kw. |
| 7 | (tomogra* or helix or helical or spiral* or spiro*).ti,ab,ot,kw. |
| 8 | ((low* adj3 dos*) or LDCT).ti,ab,kw,ot. |
| 9 | ((ultralow* or ultra-low*) adj3 dos*).ti,ab,kw,ot. |
| 10 | (low-dos* or ultralow-dos*).ti,ab,kw,ot. |
| 11 | (randomized controlled trial.pt. or controlled clinical trial.pt. or randomized.ti,ab. or randomly.ti,ab. or trial.ti,ab.)/ not (animals.mh.) not (humans.mh. and animals.mh.)) |
| 12 | (1 or 2 or 3) and (4 or 5 or 6 or 7) and (8 or 9 or 10) and 11 |
