# Supplementary material for: Effects of low-dose computed tomography on lung cancer screening: a systematic review, meta-analysis, and trial sequential analysis
Source: BMC Pulm Med. 2019 Jul 11;19:126. doi: 10.1186/s12890-019-0883-x (PMC6625016; doi:10.1186/s12890-019-0883-x)
Supplement: Supplementary file 2 — Table S2. Excluded studies with reasons. (DOCX 18 kb) [file 12890_2019_883_MOESM2_ESM.docx]

**Table S2.** Excluded studies with reasons

| No. | Citation | Reasons |
| --- | --- | --- |
| 1 | Shen H. Low-dose CT for lung cancer screening: opportunities and challenges. Front Med. 2018; **12**: 116-21. | review articles |
| 2 | Hoffmann H, Heussel CP, Eichhorn M. [Screening for Lung Cancer: Current Status]. Zentralbl Chir. 2017; **142**: S11-s6. | review articles |
| 3 | Tanoue LT. Lung cancer screening. Curr Opin Pulm Med. 2016; **22**: 327-35. | review articles |
| 4 | Al-Ayoubi AM, Flores RM. Lung cancer screening: did we really need a randomized controlled trial? Eur J Cardiothorac Surg. 2016; **50**: 29-33. | review articles |
| 5 | Zompatori M, Mascalchi M, Ciccarese F, Sverzellati N, Pastorino U. Screening for lung cancer using low-dose spiral CT: 10 years later, state of the art. Radiol Med. 2013; **118**: 51-61. | review articles |
| 6 | Kasenda B, Raatz H, Bucher HC. [Lung cancer screening - an overview about chances and risks]. Ther Umsch. 2013; **70**: 237-43; discussion 44. | review articles |
| 7 | Russi E. Lung cancer screening has the potential to safe lives, but shall we do it? Swiss Med Wkly. 2011; **141**: w13185. | review articles |
| 8 | Sagawa M, Usuda K, Aikawa H, Machida Y, Tanaka M, Ueno M, Sakuma T. Lung cancer screening and its efficacy. Gen Thorac Cardiovasc Surg. 2009; **57**: 519-27. | review articles |
| 9 | Lopes Pegna A, Picozzi G. Lung cancer screening update. Curr Opin Pulm Med. 2009; **15**: 327-33. | review articles |
| 10 | Black WC. Computed tomography screening for lung cancer: review of screening principles and update on current status. Cancer. 2007; **110**: 2370-84. | review articles |
| 11 | Grosu HB, Eapen GA, Jimenez CA, Morice RC, Ost D. Lung cancer screening: making the transition from research to clinical practice. Curr Opin Pulm Med. 2012; **18**: 295-303. | review articles |
| 12 | Field JK, Duffy SW, Baldwin DR, Brain KE, Devaraj A, Eisen T, Green BA, Holemans JA, Kavanagh T, Kerr KM, Ledson M, Lifford KJ, McRonald FE, Nair A, Page RD, Parmar MK, Rintoul RC, Screaton N, Wald NJ, Weller D, Whynes DK, Williamson PR, Yadegarfar G, Hansell DM. The UK Lung Cancer Screening Trial: a pilot randomised controlled trial of low-dose computed tomography screening for the early detection of lung cancer. Health Technol Assess. 2016; **20**: 1-146. | no relevant outcomes |
| 13 | Blanchon T, Brechot JM, Grenier PA, Ferretti GR, Lemarie E, Milleron B, Chague D, Laurent F, Martinet Y, Beigelman-Aubry C, Blanchon F, Revel MP, Friard S, Remy-Jardin M, Vasile M, Santelmo N, Lecalier A, Lefebure P, Moro-Sibilot D, Breton JL, Carette MF, Brambilla C, Fournel F, Kieffer A, Frija G, Flahault A. Baseline results of the Depiscan study: a French randomized pilot trial of lung cancer screening comparing low dose CT scan (LDCT) and chest X-ray (CXR). Lung Cancer. 2007; **58**: 50-8. | no relevant outcomes |
| 14 | Zhou Q, Fan Y, Wang Y, Qiao Y, Wang G, Huang Y, Wang X, Wu N, Zhang G, Zheng X, Bu H, Li Y, Wei S, Chen L, Hu C, Shi Y, Sun Y. [China National Lung Cancer Screening Guideline with Low-dose Computed Tomography (2018 version)]. Zhongguo Fei Ai Za Zhi. 2018; **21**: 67-75. | guidelines |
| 15 | Humphrey LL, Deffebach M, Pappas M, Baumann C, Artis K, Mitchell JP, Zakher B, Fu R, Slatore CG. Screening for lung cancer with low-dose computed tomography: a systematic review to update the US Preventive services task force recommendation. Ann Intern Med. 2013; **159**: 411-20. | guidelines |
| 16 | Sullivan FM, Farmer E, Mair FS, Treweek S, Kendrick D, Jackson C, Robertson C, Briggs A, McCowan C, Bedford L, Young B, Vedhara K, Gallant S, Littleford R, Robertson J, Sewell H, Dorward A, Sarvesvaran J, Schembri S. Detection in blood of autoantibodies to tumour antigens as a case-finding method in lung cancer using the EarlyCDT(R)-Lung Test (ECLS): study protocol for a randomized controlled trial. BMC Cancer. 2017; **17**: 187. | protocol designs |
| 17 | Quaife SL, Ruparel M, Beeken RJ, McEwen A, Isitt J, Nolan G, Sennett K, Baldwin DR, Duffy SW, Janes SM, Wardle J. The Lung Screen Uptake Trial (LSUT): protocol for a randomised controlled demonstration lung cancer screening pilot testing a targeted invitation strategy for high risk and 'hard-to-reach' patients. BMC Cancer. 2016; **16**: 281. | protocol designs |
| 18 | Sagawa M, Nakayama T, Tanaka M, Sakuma T, Sobue T. A randomized controlled trial on the efficacy of thoracic CT screening for lung cancer in non-smokers and smokers of <30 pack-years aged 50-64 years (JECS study): research design. Jpn J Clin Oncol. 2012; **42**: 1219-21. | protocol designs |
| 19 | Taylor KL, Hagerman CJ, Luta G, Bellini PG, Stanton C, Abrams DB, Kramer JA, Anderson E, Regis S, McKee A, McKee B, Niaura R, Harper H, Ramsaier M. Preliminary evaluation of a telephone-based smoking cessation intervention in the lung cancer screening setting: A randomized clinical trial. Lung Cancer. 2017; **108**: 242-6. | smoking cessation programs |
| 20 | Pineiro B, Simmons VN, Palmer AM, Correa JB, Brandon TH. Smoking cessation interventions within the context of Low-Dose Computed Tomography lung cancer screening: A systematic review. Lung Cancer. 2016; **98**: 91-8. | smoking cessation programs |
| 21 | Clark MM, Cox LS, Jett JR, Patten CA, Schroeder DR, Nirelli LM, Vickers K, Hurt RD, Swensen SJ. Effectiveness of smoking cessation self-help materials in a lung cancer screening population. Lung Cancer. 2004; **44**: 13-21. | smoking cessation programs |
| 22 | Oken MM, Hocking WG, Kvale PA, Andriole GL, Buys SS, Church TR, Crawford ED, Fouad MN, Isaacs C, Reding DJ, Weissfeld JL, Yokochi LA, O'Brien B, Ragard LR, Rathmell JM, Riley TL, Wright P, Caparaso N, Hu P, Izmirlian G, Pinsky PF, Prorok PC, Kramer BS, Miller AB, Gohagan JK, Berg CD. Screening by chest radiograph and lung cancer mortality: the Prostate, Lung, Colorectal, and Ovarian (PLCO) randomized trial. JAMA. 2011; **306**: 1865-73. | screening groups didn't include LDCT |
| 23 | Melamed MR. Lung cancer screening results in the National Cancer Institute New York study. Cancer. 2000; **89**: 2356-62. | screening groups didn't include LDCT |
| 24 | Marcus PM, Bergstralh EJ, Fagerstrom RM, Williams DE, Fontana R, Taylor WF, Prorok PC. Lung cancer mortality in the Mayo Lung Project: impact of extended follow-up. J Natl Cancer Inst. 2000; **92**: 1308-16. | screening groups didn't include LDCT |
| 25 | Sladden MJ, Ward JE. Do Australian family physicians screen smokers for lung cancer? Chest. 1999; **115**: 725-8. | doctors’ behavior |
| 26 | Brochu B, Beigelman-Aubry C, Goldmard JL, Raffy P, Grenier PA, Lucidarme O. [Computer-aided detection of lung nodules on thin collimation MDCT: impact on radiologists' performance]. J Radiol. 2007; **88**: 573-8. | impact on new technique |
| 27 | Pyenson BS, Sander MS, Jiang Y, Kahn H, Mulshine JL. An actuarial analysis shows that offering lung cancer screening as an insurance benefit would save lives at relatively low cost. Health Aff (Millwood). 2012; **31**: 770-9. | actuarial study |
