# Supplementary material for: Effects of low-dose computed tomography on lung cancer screening: a systematic review, meta-analysis, and trial sequential analysis
Source: BMC Pulm Med. 2019 Jul 11;19:126. doi: 10.1186/s12890-019-0883-x (PMC6625016; doi:10.1186/s12890-019-0883-x)
Supplement: Supplementary file 3 — Figure S1. Trial sequential analysis for lung cancer mortality. (DOCX 28 kb) [file 12890_2019_883_MOESM3_ESM.docx]

**Additional file Figure S1.** Trial sequential analysis for lung cancer mortality


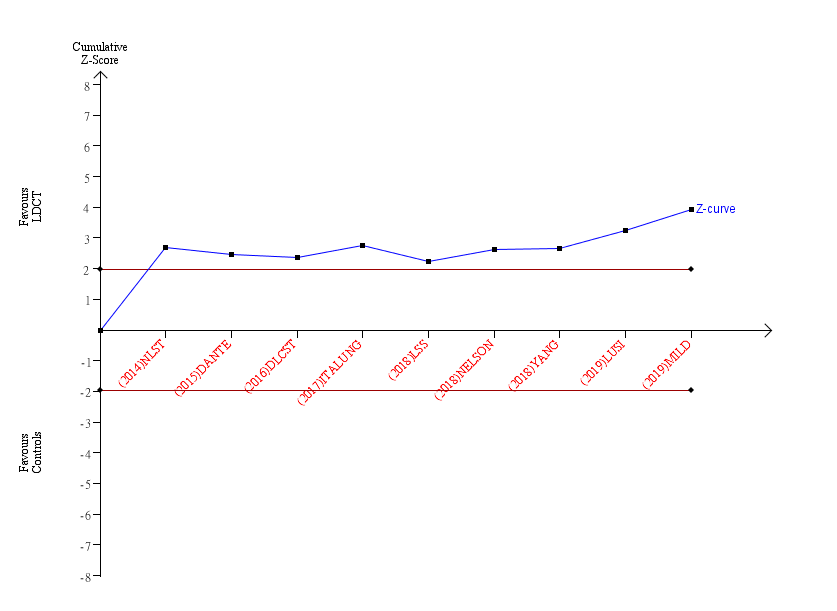


The required information size (RIS) was not renderable because the first information fraction exceeded 100% of the RIS. The Z curve has crossed the conventional boundary for benefit and RIS, indicating that the conclusion is sufficient and no more trials are needed.
